# Minimally Invasive Autopsy: A New Paradigm for Understanding Global Health?

**DOI:** 10.1371/journal.pmed.1002173

**Published:** 2016-11-22

**Authors:** Peter Byass

**Affiliations:** 1 WHO Collaborating Centre for Verbal Autopsy, Epidemiology & Global Health, Dept. of Public Health & Clinical Medicine, Umeå University, Umeå, Sweden; 2 MRC-Wits Rural Public Health and Health Transitions Research Unit (Agincourt), School of Public Health, Faculty of Health Sciences, University of the Witwatersrand, Johannesburg, South Africa

## Abstract

Peter Byass reflects on the potential niche for minimally invasive autopsies in determining cause-of-death in low- and middle-income countries.

Public health visionaries have long sought good data on mortality patterns in communities. John Graunt published his “Bills of Mortality” for the city of London in the mid-17th century (Fig 1). By the mid-18th century, Pehr Wargentin had instituted national civil registration in Sweden and published mortality analyses [1]. Nevertheless, in the 21st century, mortality patterns for the majority of the world’s population remain unrecorded, representing a major constraint on global health and development [2]. Now, Bill Gates has taken up this challenge through a Bill & Melinda Gates Foundation (BMGF) project encouraging the development of minimally invasive autopsy (MIA) to fill this gap [3].

**Fig 1:**
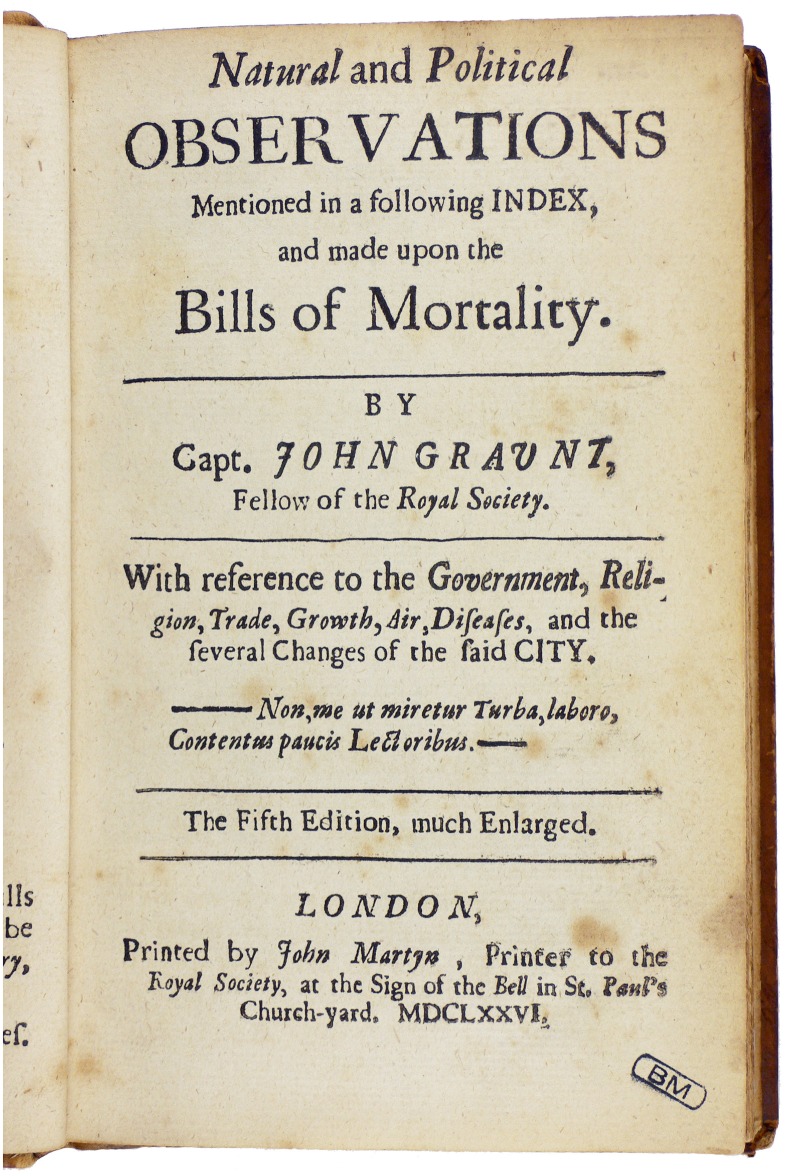
“Bills of Mortality” for London, published by John Graunt in 1676. (Public domain image from the Mansutti Foundation, https://commons.wikimedia.org/wiki/File:Graunt_-_Natural_and_political_observations,_1676_-_204.tif).

Current developments in MIA (sometimes also referred to as minimally invasive tissue sampling, or MITS) are the focus of two new research articles published this week in *PLOS Medicine* that are a part of a wider project funded by the BMGF. As with any new approach in medical science, enthusiasm for the MIA concept has to be tested against criteria for validity, feasibility, acceptability, and cost-effectiveness before MIA can be considered as a mainstream tool. Research articles from the project will seek to address these criteria, starting with an assessment by Castillo and colleagues on the validity of MIA among adults in Mozambique [4]. Although this was a relatively small study (112 cases) in one location, an encouraging degree of agreement between MIA and conventional autopsies was found. A qualitative study from Maixenchs and colleagues investigated how people regarded the hypothetical possibility of undertaking MIAs and getting feedback on causes of death in Gabon, Kenya, Mali, Mozambique, and Pakistan [5]. Although discussing hypothetical procedures may not necessarily reflect feelings that could arise about conducting actual MIAs, nevertheless, the possibility of MIAs was not regarded unfavourably. Future papers will examine other relevant aspects of MIA implementation.

## Where Does MIA Fit among Cause-of-Death Methods?

MIA enters the domain of cause-of-death assignment as a new concept among established approaches. A full postmortem examination of a body, often called an autopsy (etymologically “to see for oneself”), is often assumed to be the gold standard for determining cause of death, even though pathology findings frequently differ from clinical diagnoses [6]. There is substantial evidence that full autopsies can reveal otherwise unavailable information about a death, but families are not always comfortable with having an autopsy performed [7]. However, noninvasive (e.g., radiological) techniques may also sometimes reveal disease processes not found in an autopsy [8]. The majority of the world’s cause-of-death assignments are made by physicians issuing a death certificate, in some cases with minimal knowledge of the patient and the final illness and with consequently variable validity, particularly in relation to specific infectious aetiologies. Causes like “old age” or “heart failure” may be factual but are not epidemiologically informative. In locations where physician certificates are not mandatory, typically in low- and middle-income countries, verbal autopsy (undertaking a lay interview with appropriate informants and processing data to assign cause of death) can be used as a low-cost approach to determining cause of death. WHO has led the development of international standards for verbal autopsy, though it remains a relatively imprecise approach [9].

## How Realistic Is MIA as a Field Technique?

As the field of MIA develops, standard procedures to be followed are still a matter of discussion. For deaths occurring in hard-to-reach areas of low- and middle-income countries, the inclusion of procedures requiring extensive infrastructure, such as advanced radiology, may be impossible. Thus, MIA protocols relying principally on needle sampling have been proposed for low- and middle-income countries [10]. Whether this approach will be adequate for tracking the increasing incidence of deaths from noncommunicable diseases in low-resource settings remains as an important question to answer.

The potential niche for MIA in the cause-of-death landscape needs to be considered carefully. In low- and middle-income countries, full autopsies are very rare and unlikely to be feasible at all in rural areas, due to both resource constraints and acceptability. MIA involves invasive interactions with a recently dead body, which involves logistic, technical, and cultural challenges, even if it may be much more feasible than a full autopsy. Building up practical experience of the MIA process across a wide range of settings, including the whole age range from stillbirths to older adult deaths, different cultures and religions, and urban and rural communities, will be essential in defining best practice and determining what may be acceptable. Equally, yields of useful biomedical information in relation to sampling methods and diagnostic procedures used will need to be examined carefully in order to develop guidelines for best practice and cost-effectiveness. Oversampling in a MIA examination is likely to hugely increase costs, particularly in relation to preserving, transporting, and analysing samples, so optimal practices need to be carefully determined.

## What Might MIA Add to Understanding Population Health?

Whether MIA will eventually become a widely used routine practice remains a moot point at this stage. Even if undertaking a MIA exam were to cost less than, say, US$1,000 in a more routine context, the expenditure could still represent a considerable opportunity cost in relation to costs of routine preventive actions such as vaccination. But there may also be another scenario, one in which limited series of MIA exams undertaken in conjunction with carefully conducted verbal autopsy interviews might contribute to an international reference database. One of the difficulties faced by verbal autopsy methods is the lack of definitive connections between characteristics of final illnesses and specific causes of death, even within data specifically gathered for this purpose [11]. If MIA with verbal autopsy were able to determine particular associations between symptoms and aetiologies leading to death, such data (on a sufficiently large scale) could be hugely informative for improving future verbal autopsy standards and models. That in turn could make large-scale and reliable cause-of-death assignment much more viable and cost-effective.

MIA shows signs of being an important addition to the world’s available range of cause-of-death assignment methods. Exactly how useful it may turn out to be in the long run will depend very much on findings from detailed methodological evaluations such as those reported in this week’s *PLOS Medicine* and the wider collection of papers that will emerge from the project.

## Abbreviations

BMGF: Bill & Melinda Gates Foundation
MIA: minimally invasive autopsy
MITS: minimally invasive tissue sampling
